# Performance Prediction Equation for 2000 m Youth Indoor Rowing Using a 100 m Maximal Test

**DOI:** 10.3390/biology10111082

**Published:** 2021-10-22

**Authors:** Luiz Felipe da Silva, Paulo Francisco de Almeida-Neto, Dihogo Gama de Matos, Steven E. Riechman, Victor de Queiros, Joseane Barbosa de Jesus, Victor Machado Reis, Filipe Manuel Clemente, Bianca Miarka, Felipe J. Aidar, Paulo Moreira Silva Dantas, Breno Guilherme de Araújo Tinoco Cabral

**Affiliations:** 1 Health Sciences Center, Department of Physical Education, Federal University of Rio Grande do Norte, Natal 59078-970, Brazil; paulo220911@hotmail.com (P.F.d.A.-N.); victorsabino97@ufrn.edu.br (V.d.Q.); pgdantas@icloud.com (P.M.S.D.); brenotcabral@gmail.com (B.G.d.A.T.C.); 2 Cardiorespiratory & Physiology of Exercise Research Laboratory, Faculty of Kinesiology and Recreation Management, University of Manitoba, Winnipeg, MB R3T 2N2, Canada; dihogogmc@hotmail.com; 3 Department of Health and Kinesiology, Texas A&M University, College Station, TX 77843, USA; sriechman@tamu.edu; 4 Group of Studies and Research of Performance, Sport, Health and Paralympic Sports GEPEPS, The Federal University of Sergipe, UFS, São Cristovão 49100-000, Brazil; josybj@hotmail.com (J.B.d.J.); fjaidar@academico.ufs.br (F.J.A.); 5 Graduate Program in of Physical Education, Federal University of Sergipe—UFS, São Cristovão 49100-000, Brazil; 6 Research Center in Sports Sciences, Health Sciences, and Human Development (CIDESD), Trás os Montes and Alto Douro University, 5001-801 Vila Real, Portugal; victormachadoreis@gmail.com; 7 Sports and Leisure, Polytechnic Institute of Viana do Castelo, Rua Industrial and Commercial School of Nun’Álvares, 4900-347 Viana do Castelo, Portugal; filipe.clemente5@gmail.com; 8 Telecommunications Institute, Delegation of Covilhã, 1049-001 Lisbon, Portugal; 9 Laboratory of Psychophysiology and Performance in Sports & Combats, Postgraduate Program in Physical Education, School of Physical Education and Sport, Federal University of Rio de Janeiro, Rio de Janeiro 21941-901, Brazil; miarkasport@hotmail.com; 10 Department of Physical Education, Federal University of Sergipe—UFS, São Cristovão 49100-000, Brazil; 11 Program of Physiological Science, Federal University of Sergipe—UFS, São Cristovão 49100-000, Brazil

**Keywords:** athletic performance, rowing, sport, young athlete, mathematical model

## Abstract

**Simple Summary:**

The 2000 m tests, usually applied in indoor rowing, during weeks of evaluation and selection of young rowing athletes, often discourage participation or are performed by athletes without a previously established strategy (i.e., execution strategy, according to an estimated performance expectation) which may underestimate the performance of young athletes. Thus, the mathematical model developed in this research can contribute to the selection of athletes in Olympic rowing by providing a low-cost tool with a significant level of reliability and performance prediction of 2000 m. Furthermore, the mathematical model could help to propose highly reliable assessment strategies following coaches. This model could be used as an alternative to traditional ways of evaluating training progression up to 2000 m, thus contributing to the strategic planning of the tests applied and the development of athletes.

**Abstract:**

Background: The exhaustive series of tests undergone by young athletes of Olympic rowing prior to important competitions imply loads of physical stress that can ultimately impact on mood and motivation, with negative consequences for their training and performance. Thus, it is necessary to develop a tool that uses only the performance of short distances but is highly predictive, offering a time expectancy with high reliability. Such a test must use variables that are easy to collect with high practical applicability in the daily routine of coaches. Objective: The objective of the present study was to develop a mathematical model capable of predicting 2000 m rowing performance from a maximum effort 100 m indoor rowing ergometer (IRE) test in young rowers. Methods: The sample consisted of 12 male rowing athletes in the junior category (15.9 ± 1.0 years). A 100 m time trial was performed on the IRE, followed by a 2000 m time trial 24-h later. Results: The 2000 m mathematical model to predict performance in minutes based on the maximum 100 m test demonstrated a high correlation (r = 0.734; *p* = 0.006), strong reliability index (ICC: 0.978; IC95%: [0.960; 0.980]; *p* = 0.001) and was within usable agreement limits (Bland -Altman Agreement: −0.60 to 0.60; 95% CI [−0.65; 0.67]). Conclusion: The mathematical model developed to predict 2000 m performance is effective and has a statistically significant reliability index while being easy to implement with low cost.

## 1. Introduction

Olympic Rowing is characterized by a high physical demand in which a high aerobic and anaerobic capacity are required for optimal performance [1,2]. Depending on the athletes’ gender and age and the type of the boat, a 2000 m Olympic rowing race can last from 5 to 8 min [3,4]. During a rowing race, the metabolic source of energy is predominantly aerobic [3,4]. Yet, both in the first ~100-m and at the last ~200-m race, athletes tend to perform maximal output sprints that can be decisive at the finish line [3,4]. Those sprints require a rapid and high load of metabolic energy, with the anaerobic system taking over. Therefore, a significant contribution of the anaerobic capability and efficiency of this metabolic system can also predict rowing performance [3,4]. For instance, the modified Wingate performance for rowing in national-level adolescent rowers could be used to predict rowing performance [3]. Accordingly, the anaerobic stimuli, as an indicator of the anaerobic capacity, indirectly reflects rowers’ performance at 2000 m [3,4,5,6,7]. Previous studies have suggested that parameters such as race time and short tests [i.e., 50-m, 100-m, or 500-m anaerobic stimuli performed on a rowing ergometer] should be taken into consideration during the process of selection and orientation of young athletes [5,6,7,8].

Several studies have reported a positive correlation between 2000 m indoor rowing performance and power produced during 20 s, 30 s, and 60 s rowing tests [5,6,7,8]. Cerasola et al. [8] developed a mathematical model for predicting 2000 m performance in indoor rowing using the combination of anthropometric variables, VO2max, and 60 s maximum sprint. In a study conducted by Maciejewski [9], the researchers found a strong correlation of the Wingate test adapted for rowing, with 1500 m indoor rowing performance in competitive adolescent rowers, and the results point out that rowing coaches can use the modified Wingate test to potentially identify talented young rowers. In the same study, the authors found a strong correlation of the 60 s test with 2000 m performance and point out that 60 s performance can be considered a valuable tool to predict 2000 m performance of elite young rowers, not requiring expensive and long duration [7].

Anaerobic energy pathways can be entered into regression models to predict ergometer performance at 2000 m and identify rowing talent [3]. Regression analysis models for performance prediction have already been suggested for the rowing modality [3,5,6,7]. Nonetheless, although results of studies have contributed to the scientific knowledge of rowing, few coaches use these methods due to the requirement of sophisticated equipment for evaluation, which is not always available to them. Therefore, they choose to use traditional evaluation methods in the selection of young athletes [3,5,6,7]. In addition, reliable mathematical models that use only short distance performance to predict 2000 m performance in indoor rowing are not yet available in the scientific literature [5,6,7,8,9,10]. Therefore, relating practice tests to success in rowing may be beneficial in adapting and constructing training plans to optimize athlete development [9,10]. Thus, developing specific tests for rowers with good reproducibility could help predict performance, and training progression in the training environment becomes necessary.

A mathematical model that could predict the performance and progression of training would help coaches in the initial planning of training objectives, also being useful to assess the anaerobic capacity of rowers indirectly, in addition to providing accurate information for estimation of performance concerning increasing the distance of the tests applied in 2000 m with low cost, high practical applicability, and easy collection. [7,9,10].

Sports scientists have suggested complete indoor rowing tests of 20 s and 30 s [3,5,6]. However, this assessment does not reflect the initial phases and final sprints that last about 60 s, with an estimated energy expenditure of 500–700 Watt [11,12,13]. Therefore, a maximum 100 m ergometer rowing test might be better suited to monitor rowers’ ability to sustain energy expenditure during the actual start and finish phases of a 2000 m rowing.

The present study aimed to develop a mathematical model capable of predicting 2000 m performance for young rowers from a 100 m maximal effort test. The present study raised the hypothesis that the performance of 2000 m in rowing could be predicted with a mathematical model using parameters easily collected by the coaches and reproducible in their daily environment.

## 2. Materials and Methods

### 2.1. Participants

The research has a cross-sectional design with a sample selected from February to March 2020 at the “Sports Club de Natal” (Brazil). The sample was composed of twelve young male rowing athletes (15.9 ± 1.0 years; body weight (kg): 66.5 ± 13.1; height: (cm) (170.7 ± 7.0, wingspan (cm): 161.5 ± 43.2, body mass index (kg/m^2^): 22.6 ± 3.4, time of practical experience in rowing: (1.3 ± 2.0 years), who were ranked on the national level were selected for the study. Based on criteria from Matsudo, Rivet, and Pereira [14], the sample is classified as national-level athletes. These athletes were among the top 20 in the positions between 7th and 12th place (final B) and between 13th and 19th place (final C) national ranking during 2020.

Table 1 reports the characteristics of the subjects about body composition and power during 100-m and 2000-m tests.

Inclusion criteria were: (i) Being affiliated to a state federation and national Olympic rowing confederation; (ii) at least one year of training experience; (iii) age 14 to 16 years, and; (iv) a minimum training frequency of six times a week (≥60 min per training session)

Exclusion criteria were: (i) Presence of osteoarticular lesions (i.e., lesions in bone tissues and joints) or muscle injuries in the last six months before the research. (ii) could not complete all the tests proposed by this study. (iii) use of ergogenic substances (i.e., supplements) that could enhance physical performance (i.e., caffeine, taurine, creatine, and others).

The Ethics approved this study, and Research Committee with Human Beings of the Federal University of Rio Grande do Norte (technical advice: 3.552.010), respecting the national and international ethical principles in the declaration Helsinki and ethical standards in sport and exercise science research [15]. In addition, the present study complies with items on the STROBE checklist for observational studies [16]. All participants and their guardians received information about the research objectives and methodological procedures adopted. Informed consent terms (TALE) and a free and informed consent term (ICF) were signed by the volunteers and their respective legal guardians, according to Resolution 466/12.

### 2.2. Procedures

**Figure 1:**
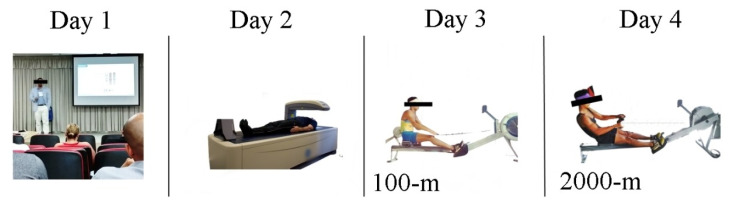
Study design.

On the first day, all procedures for the experiment were explained to participants and their respective guardians. On the second day, anthropometric and body composition tests were performed for sample characterization purposes. On the third day, the athletes performed the 100 m sprint. Finally, on day 4, a 2000 m time trial was performed (See Figure 1). All rowing testing was conducted on an indoor rowing ergometer (Concept^®^ model-D equipped with PM5 digital monitor; Morrisville, CT USA) [17] in a temperature-controlled environment (26 °C). The equipment was calibrated with a resistance factor of 125 (N s^2^/m^2^) (i.e., Air System specific to Indoor Rowing) according to the specifications of the international rowing federation. All tests were performed on consecutive days starting at 8:00 am.

### 2.3. Analysis of Body Morphology

For sample characterization, body composition was determined with dual-energy X-ray absorptiometry (DEXA) (LUNAR®/GE PRODIGY—LNR 41,990, Software enCORE version 18; United States–Washington DC) using specific algorithms for the pediatric population [18].

### 2.4. Development of the Mathematical Model

A mathematical model was developed to predict 2000 m performance based on the performance of the maximum effort 100 m Indoor Rowing Test with consideration of a priori theoretical model [19,20,21]. Thus, based on traditional physics, we developed a mathematical model of approaching the distance from the sprint time of 100 m (in seconds) [19,20,21]. In this sense, we consider the time in seconds of the sprint of 100 m multiplied by constant 22 (number determined by algorithms for an approximation with the time of 2000 m). Subsequently, the correction factor +18 was identified by algorithms to equalize the results of the predictive equation with those of indoor rowing. Thus, to convert the results into minutes, we divide the final product by 60 [19,20,21]. Subsequently, regression analyses were performed, in sequence and the theoretical model was tested using confirmatory factor analyses and the reproducibility index [19].

The following is the mathematical model developed to predict 2000 m performance from the performance in the 100-m test:

Time in Minutes 2000 m = [(Time in Seconds 100 m × 22) + 18]/60

### 2.5. Statistics

To determine a priori the minimum sample size to develop the mathematical model, we used the effect size of 0.915, referring to the linear regression result (mathematical model for peak power X peak power in indoor rowing) found by Almeida-Neto et al. [22]. We used the G*Power ^®^ software (Version 3.0; Berlin, Germany) in the configuration “T family statistics for regressions” and an α = 0.05 and a β = 0.80 considering a single variable to perform the prediction. A minimum sample size of 10 subjects (t (2.0) = 2.91) was determined to be an acceptable sample size with power estimated to be 0.90. The normality of the data was analyzed by the Shapiro-Wilk tests and z-score for asymmetry and kurtosis (−1.96 to 1.96).

Pearson’s linear correlation test determined the data correlations. The correlational magnitude thresholds used were those proposed by: Insignificant: r < 0.10; Weak: r = 0.10–0.39; Moderate: r = 0.40–0.69; Strong: r = 0.70–0.89; Very strong: r = 0.90–1.00 [23]. The Breush-Pegan test tested the homogeneity of the models, and the assumptions of normality, variance, and independence of the data were confirmed. The Durbin–Watson test was used to test the multicollinearity of the regression models. By White’s test, we checked the heteroscedasticity of the regression models.

To measure the reproducibility and reliability of the mathematical model, an analysis of the intraclass correlation coefficient was performed, with magnitude of absence: ICC ≤ 0; poor: ICC = 0–0.19; weak: ICC = 0.20–0.39; moderate: ICC = 0.30–0.59; substantial: ICC = 0.60–0.79; and almost complete: ICC ≥ 0.80 [24]. The Bland-Altman method was used to verify the degree of agreement between the models. By proportion bias analysis, we checked for heteroscedasticity of the Bland-Altman concordance.

For the comparative analysis, the Student t-test was used. The size of the effect of the differences was calculated by the Cohen test (d). The magnitude of the Effect Size followed the classification described by Espírito Santo and Daniel [25]: insignificant <0.19; 0.20–0.49 small; mean 0.50–0.79; large 0.80–1.29; very large <1.30). For the technical error of anthropometric measurements, the following magnitude was used: Acceptable for skin folds ≤ 5.0% and other anthropometric measurements ≤1.0% [26]. All analyses were performed using open-source software R (version 4.0.1; R Foundation for Statistical Computing^®^, Vienna, Austria) with a significance threshold of *p* < 0.05.

## 3. Results

The 100 m (seconds) performance correlated significantly with the 2000 m (minutes) performance. The linear regression model in Table 2 shows that the 100 m performance was also efficient in predicting the 2000 m performance. We emphasize that for the linear regression, no significant heteroscedasticity or significant multicollinearity was detected.

The G * Power software (Version 3.0; Berlin, Germany) was used to check the power of the post-hoc results (Table 2), and in the “T” statistic configuration for correlations and regressions, reporting the effect as r^2^ (see Table 2), adopting α = 0.05. Thus, for the correlation analysis, a sampling power of 0.950 (t (10.0) = 1.75) and regression of 0.960 (t (11.0) = 1.80) suggests that the findings of the present study are reliable.

**Figure 2:**
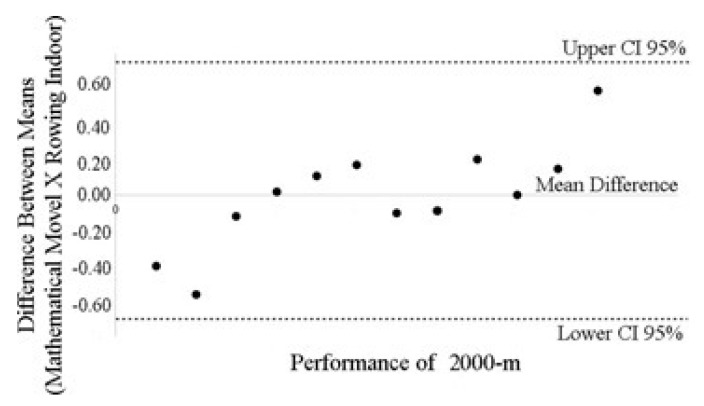
Bland-Altman Plot. CI 95%: Confidence Interval of 95%. M: Meters.

The only variable used in the model was the 100 m sprint test time in seconds. In addition, the result predicted by the mathematical model showed a substantial reliability index and a significant agreement index (see Figure 2), with the result of the 2000 m indoor rowing performance (CCI = 0.978; IC95%: [0.960; 0.980]; *p* = 0.001); (Bland-Altman Agreement: −0.60 to 0.60; IC 95%: [−0.65; 0.67]). Figure 2 shows the limits of agreement of the performance in minutes predicted by the mathematical model with the actual performance in minutes. No significant heteroscedasticity was observed, and the difference between the methods, was values between −0.60-min and 0.60-min (the differences ranging between 7% and 11%).

**Figure 3:**
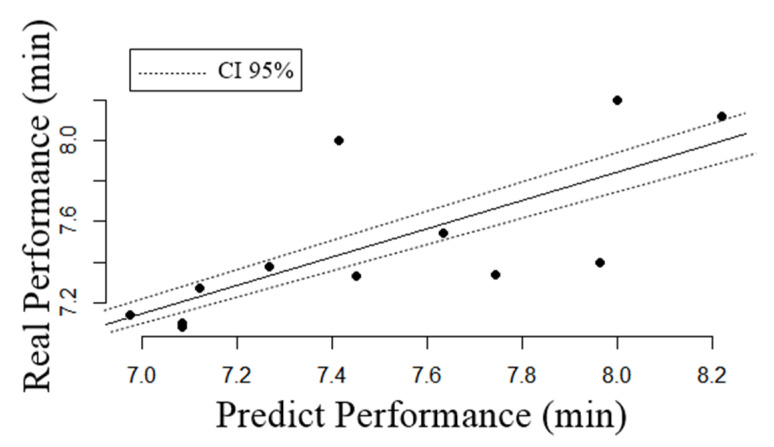
Comparison of the result predicted by the mathematical model with the result of the 2000 m test in indoor rowing. CI 95%: Confidence Interval of 95%. (min): Minutes.

Figure 3 shows that no significant differences were observed when comparing the result of the 2000 m test performed on an indoor ergometer with the result of the mathematical model developed presently (Real Performance (minutes): 7.49 ± 0.39, Coefficient of variation: 5.2%, standard error: 0.11; Predicted performance (minutes): 7.50 ± 0.41 Coefficient of variation: 5.4%, standard error: 0.12; Effect Size = 0.01; IC 95%: [−0.83; 0.85]; *p* = 0.9). It should be noted that the mathematical model showed no significant bias in relation to the performance of 2000 m (difference between the methods = 0.00 ± 0.30; r^2^ = 0.094; β = −1.72, CI 95% β: [−5.02; 0.09]; *p* = 0.33), which suggests that there is no systematic bias in the model developed by this study. However, based on the individual data, the mathematical model points out the limitation of underestimating or overestimating the 2000 m time by ~12-s in 7 out of 12 athletes (58%).

## 4. Discussion

The present study aimed to develop a mathematical model to predict rowing performance at a distance of 2000 m in an indoor maximum rowing test at a distance of 100 m. This study pioneered the use of a 100 m top speed test to predict 2000 m rowing performance in young males. The main finding of this study demonstrates that the mathematical model based on a maximum effort of 100 m was moderately significant to predict the performance of 2000 m in indoor rowing. However, the mathematical model of the present study underestimated or overestimated the 2000-m time of 58% of athletes in ~12 s.

Previously, in a study conducted by Šmída et al. [27], a significant relationship was observed between the peak anaerobic power and 2000 m indoor rowing performance. This result suggests that a test with a predominant anaerobic energy requirement could be a good predictor of aerobic performance in the 2000 m rowing test [27]. It is known that in rowers, the energy demands of a standard test of 2000 m are predominantly aerobic. However, one-third and a quarter of the total energy demand comes from anaerobic sources [28,29]. Due to the high resistance and physical strength required by this modality, both energetic (i.e., aerobic and anaerobic) pathways end up being stressed submaximally or maximally [30].

In a similar study, Cataldo et al. [5] developed a 2000 m rowing performance prediction model in 20 young male athletes (average age 15.2 ± 1.3) and concluded that the 2000 m rowing performance could be estimated from a 20 s indoor rowing sprint test. However, this mathematical model requires the use of sophisticated and costly testing, such as fat-free mass index and maximum VO2 (VO2max) levels consumed during the 20-s sprint. The mathematical model created by Riechman et al. [6] proved to effectively predict 2000 m performance in highly trained young women from a maximum 30 s sprint performance (Rowing Wingate). Riechman et al. [6] reported statistically significant correlations in the range of r = 0.84 to r = 0.89 between the results of the Wingate anaerobic rowing test and the performance of the 2000 m ergometer rowing test. The significant relationship between the Wingate test results and the performance of the 2000-m rowing ergometer is likely to be explained, in part, by a substantial aerobic contribution to a 30 s test [6]. However, this study used the mean power variables of 30 s maximum, VO2max, and fatigue percentage of the Wingate test. Therefore, the equations developed by Cataldo et al. [5] and Riechman et al. [6] require a sophisticated laboratory evaluation before using the 2000 m performance prediction equation.

Given this assumption, it is suggested that the time in seconds of a short performance in rowers is a reliable predictor of competition distance performances [28,29,30], which eliminates the need to perform sophisticated analyses such as those mentioned above. Thus, the mathematical model presented in this study used only the 100 m sprint time to estimate the 2000 m performance, with significant reliability. The use of the 100-m test becomes practical for the rower training environment, being a significant anaerobic stimulus that can help in predicting the 2000-m challenge. Billat et al., [31] point out that anaerobic capacity is predictive of rowing performance, attributing this fact to sprints often performed during rowing race. Thus, the results of the present study indicate moderately significant relative reliability. The coefficient of variation values was low (<6%), as well as the standard error values (<0.15) and the difference in relation to the base method (indoor rowing) were less than 1%, demonstrating that the mathematical model based in one sprint of 100-m, can be helpful for sports. According to Atkinson and Nevill [32], the estimate of performance in sports needs to have high reliability and an agreement above 95% in relation to the basic method used. Thus, the present study showed a significant reliability index (ICC: 0.978; IC95%: [0.960; 0.980]; *p* = 0.001) and thus can contribute to the selection of athletes in Olympic rowing by providing a low-cost tool with a significant level of applicability and prediction of 2000 m performance.

Seeking to propose a highly reliable assessment strategy for rowing coaches, the strength of this study was to present an equation developed as an effective tool to predict the athlete’s performance at a distance of 2000 m. Therefore, this research can contribute to the monitoring and evaluation of young rowers, providing a tool with a significant level of reliability to predict 2000 m performance. In addition, our equation may be an alternative to traditional ways of evaluating training progression for the 2000 m, thus contributing to planning and development parameters for athletes and coaches. Therefore, this research can contribute to the monitoring and evaluation of young rowers, providing a tool with a significant level of reliability to predict 2000 m performance.

The strengths of the present research were: (i) the study design was adequate to an-swer the research question by presenting an assessment model with high reliability; (ii) gender was not a divergent factor in the current sample as we used only male athletes; (iii) Significant practical applicability for rowing coaches as it was easy to use and repeated frequently due to its short duration.

Despite the relevance of the results, this study is limited by the fact that it was per-formed only with a small number of young male athletes, and athletes from other catego-ries, ages, and sex may present different results. In addition, we highlight that although the mathematical model has practical applicability, its predicting power is limited and the absence of comparisons with more sophisticated mathematical models that use variables such as VO2max [5,33,34] is also a limitation to accurately determine the quality of the mathematical model developed by the present study. In addition, the mathematical model points to an estimation error of ~12-s in 58% of the sample (underestimating or overestimating). This suggests that a single stimulus of 100-m should not be the only criteria used to evaluate rowers. To improve the model, it may be necessary to use more physiological variables (i.e., heart rate, cardiorespiratory capacity, lactate threshold, etc.) or non-physiological variables (i.e., body composition, rowing power, etc.) that correlate with the performance of 2000-m.

This, results suggest that future studies seek to improve the mathematical model of the present study, increasing physical fitness tests that are easy to use in the coaches’ work environment. Therefore future studies that seek to demonstrate the reliability of the mathematical model of the present study with inclusion of metabolic variables from indirect and direct methods are encouraged.

## 5. Conclusions

The results of this study show that the predictive equation proposed for performance of 2000 m is moderately reliable and predicts performance within 5% of actual performance. Thus, the equation model presented is low cost, and favors time savings and lower physical wear for athletes. It is necessary that future studies improve the mathematical model in order to provide a tool with lower estimation errors, thus providing an option to evaluate the performance prediction and monitor training using a maximum 100-m indoor rowing test.

## Figures and Tables

**Table 1:** Subject Characteristics.

Variables	Mean ± SD
Fat mass (kg)	16.5 ± 6.7
Lean mass (kg)	47.4 ± 8.1
Mean Power in 100 m (watts)	376.9 ± 62.7
Mean Power in 2000 m (watts)	235.9 ± 29.0

kg = kilograms. kg/m^2^ = kilograms per square meter. m = Meters. SD = Standard deviation.

**Table 2:** Correlations and regressions of variables with a performance at different distances in rowing.

Variable	Rowing 2000 m					
**Rowing 100 m**	**Correlation**			**Regression**		
	**r**	**r^2^**	***p*** **Value**	**(r^2^)**	**β**	***p*** **Value**
	0.734 *	0.538	0.006	0.539 *	15.42	0.006

* Statistically significant; r = correlation coefficient; r^2^ = squared correlation coefficient. (r^2^) = regression determination coefficient; β = angular regression coefficient in relation to the dependent variable. m = Meters.

## Data Availability

The data that support the findings of this study are available from the corresponding author upon reasonable request.
